# How does social context influence appraisal and help‐seeking for potential cancer symptoms in adults aged 50 and over? A qualitative interview study

**DOI:** 10.1111/ecc.13640

**Published:** 2022-06-21

**Authors:** Rosalind Adam, Alison J. Thornton, Katriina L. Whitaker, Peter Murchie, Philip C. Hannaford, Susan Hall, Sarah Smith, Alison M. Elliott

**Affiliations:** ^1^ Academic Primary Care, Institute of Health Sciences University of Aberdeen Aberdeen UK; ^2^ School of Health Sciences University of Surrey Surrey UK; ^3^ Graduate School Abertay University Dundee UK

**Keywords:** cancer, decision making, early detection of cancer, health‐seeking behaviours, qualitative research, social networking

## Abstract

**Objective:**

To investigate how social context and social network activation influence appraisal and help‐seeking for symptoms potentially indicative of cancer.

**Methods:**

Semi‐structured telephone interview study. Community dwelling adults who had experienced at least one symptom potentially indicative of cancer within the last month were sampled from a national symptom survey.

**Results:**

Thirty‐four interviews were conducted. Participants looked to peers and wider society to judge whether symptoms might be normal for their age. Involvement of others in symptom appraisal promoted an active management strategy, such as contacting a healthcare professional or trying a medication. There were practical, emotional, attitudinal, normative and moral barriers to involving others. Cancer narratives from significant others, public health campaigns and the media influenced symptom appraisal. Participants held mental representations of types of people who get cancer, for example, smokers and unfit people. This had two consequences. First, participants did not identify themselves as a candidate for cancer; impeding help‐seeking. Second, social judgements about lifestyle introduced stigma.

**Conclusion:**

Involving friends/family in symptom appraisal facilitates help‐seeking but barriers exist to involving others. Campaigns to promote earlier cancer diagnosis should incorporate age‐appropriate narratives, address misconceptions about ‘types’ of people who get cancer and tackle stigma about lifestyle factors.

## 1 INTRODUCTION

The patient interval (period between detecting a symptom and first presentation to a healthcare professional) forms an important component of diagnostic interval in cancer (Andersen & Cacioppo, 1995; Hansen et al., 2011). Longer diagnostic intervals are associated with more advanced cancer stage at diagnosis and poorer patient outcomes (Tørring et al., 2013,  2017).

The processes involved in detecting bodily changes, recognising a symptom as a deviation from the norm, interpreting its meaning, deciding what action to take and ultimately taking action, are non‐linear (Scott et al., 2013) and influenced by a multitude of symptom‐, individual‐, social‐ and cultural‐related factors (Andersen et al., 2010; Wyke et al., 2013).

Personal social networks can influence decisions about whether to seek help from a general practitioner (GP). A study of patients consulting GPs with new symptoms showed that 70% had conversations with others that were of importance in the decision to consult (Cornford & Cornford, 1999). Research examining help‐seeking for potential cancer symptoms has mainly focused on professional network activation (i.e., what leads individuals to consult a professional) (Niksic et al., 2015; Smith et al., 2005). Less is known about the activation and roles of personal social networks.

Though under‐researched (Macdonald et al., 2019), the role of social networks and the socio‐cultural context of cancer symptom appraisal are likely to be of considerable importance. A systematic review found that disclosing symptoms to a family member could reduce time to presentation to a healthcare professional by two to six times (McCutchan et al., 2015). Cancer alarm symptoms which become obvious to others are also more likely to lead to medical attention (Whitaker et al., 2015). Social approval of help‐seeking by others is an important driver of early presentation to a healthcare professional (Howell et al., 2008; Oberoi et al., 2016). Conversely, competing priorities (e.g., family responsibilities or employment) can impede help‐seeking (Burgess et al., 2001; Gould et al., 2010).

This study aimed to explore the experiences, cognitive processes and subsequent behaviours of individuals who had recently experienced a symptom potentially indicative of cancer. A particular focus was the investigation of how community dwelling adults approach personal social network activation after experiencing a symptom and the social/societal context in which symptom appraisal and help‐seeking occur. We considered personal social network activation to broadly include processes such as disclosing symptoms to friends, acquaintances, or significant others and/or asking for advice.

## 2 METHODS

### 2.1 Study design, sampling frame and recruitment

A theory‐based semi‐structured telephone interview study was conducted. The study population was derived from respondents to the Understanding Symptom Experiences Fully (USEFUL) study questionnaire (Hannaford et al., 2020). The questionnaire was sent to a sample of 50,000 adults aged 50 years and over, registered with 21 General Practices across Scotland and the West Midlands of England. There were 16,778 respondents.

The questionnaire asked about respondents' experiences of 25 different symptoms (Table 1) during the previous one and 12 months and what action they had taken about the symptom(s). Participants reporting symptoms within the last month were asked to indicate which one bothered them the most. Although the study had a particular interest in assessing symptoms potentially indicative of cancer, cancer was not mentioned in any of the study materials. Twenty‐one symptoms were potentially indicative of one of four common cancers and four symptoms were more general.

**TABLE 1:** Symptoms enquired about in the Understanding Symptom Experiences Fully (USEFUL) questionnaire (listed as presented in the questionnaire)

Headaches	Masking symptom
Persistent indigestion/heartburn	Upper gastrointestinal cancer associated
Difficulty swallowing	Upper gastrointestinal cancer associated
Stomach or abdominal pain	Upper gastrointestinal cancer associated
Chest pain	Lung cancer associated
Hoarseness	Lung cancer associated
Loss of appetite	Non‐specific cancer associated
Unexplained weight loss!!	Non‐specific cancer associated
Persistent cough	Lung cancer associated
Change in ongoing cough	Lung cancer associated
Persistent diarrhoea	Colorectal cancer associated
Persistent constipation	Colorectal cancer associated
Coughing up phlegm	Lung cancer associated
Coughing up blood	Lung cancer associated
Shortness of breath	Lung cancer associated
Wheezy chest	Masking symptom
Change in bladder habits	Masking symptom
Change in bowel habits	Colorectal cancer associated
Blood in stool or rectal bleeding!!	Colorectal cancer associated
Back or joint pain	Masking symptom
Persistent vomiting	Upper gastrointestinal cancer associated
Vomiting up blood	Upper gastrointestinal cancer associated
Lump in breast	Breast cancer associated
Breast change other than lump	Breast cancer associated
Tired all the time	Non‐specific cancer associated

The interview sampling frame included individuals who consented to further contact and who had experienced at least one of the 21 symptoms potentially indicative of cancer within the last month. Technical restraints with the online questionnaire response system meant that sampling was limited to those who returned their questionnaire by post (11,596 of 16,778 respondents).

Purposive sampling (Ritchie & Lewis, 2003) was used to achieve maximum variation in the sample, using age, gender, sociodemographic group (indicated by education and household income), the symptom that the respondent had rated most bothersome and whether or not the respondent had contacted their GP about the symptom in the last month.

Interviews were to be conducted until data saturation was reached—defined as at least two successive interviews in which no new themes were identified (Francis et al., 2010). Study and personnel constraints prevented concurrent data analysis and collection, so we relied on the interviewer's judgement when saturation had occurred.

### 2.2 Theoretical framework

**FIGURE 1:**
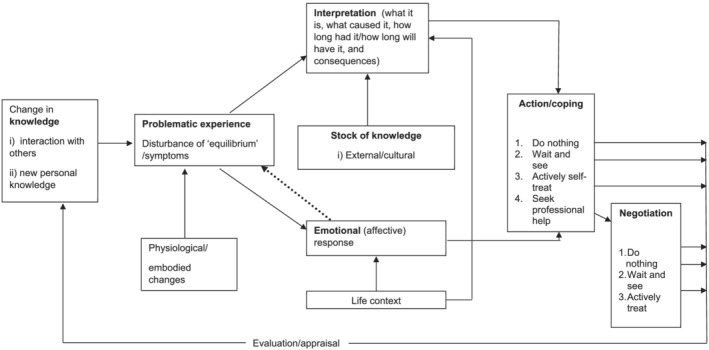
Theoretical framework on which interview schedules and data coding were based, integrating CSM, Illness Action Model and Network Episode Model

A topic guide was developed using an integrated theoretical model to ensure comprehensive coverage of the topic and to incorporate existing knowledge. A schema of the integrated model is presented in Figure 1 (Smith, 2010). It incorporates the Common‐Sense Self‐Regulation Model (CSM) (Leventhal et al., 1980), Illness Action Model (Dingwall, 2001) and Network Episode Model (Pescosolido, 1992). These models have been described in detail by Wyke et al. (2013).

Participants were asked to tell the interviewer everything they could about their main symptom and any additional symptoms they had experienced. Questions were included about symptom timeline, ideas about causes of the symptom(s), impact/consequences of the symptom(s), emotional response to the symptom(s) and coping procedures. Individuals were asked whether anyone else had influenced their thoughts or response to the symptom(s) and the influence of family/friends/health services/media, social networks, employment situation, family responsibilities, stressful events, cultural expectations and barriers/facilitators to help‐seeking associated with social context.

### 2.3 Data generation, management and analysis

AJT conducted in‐depth, semi‐structured telephone interviews. Interviews were audio‐recorded, transcribed verbatim and imported into NVivo Version 11. Four authors (RA, AJT, SH and SS) read all transcripts in detail, familiarising and immersing themselves in the data. Data were organised and categorised using a coding framework derived from the topic schedule and theoretical model. New codes were added during analysis. Data were also summarised in a framework matrix (Ritchie & Lewis, 2003) in which each participant occupied a row. Key topics were assigned a column, and participant quotations were added to the matrix. Thematic analysis (Joffe & Yardley, 2004) was conducted on coded transcripts and the framework matrix. Involvement of four authors in data analysis reduced the risk of bias.

### 2.4 Ethics

The East Midlands (Derby) ethics committee approved the study by proportionate review, reference (14/EM/1124).

## 3 RESULTS

Invitation packs were posted to 201 individuals. The response rate was 50% (101/201 consents returned). Thirty‐five telephone interviews were conducted, although one was subsequently excluded from analysis because the participant had been diagnosed with a condition that was causing difficulties with short‐term memory. The mean duration of the remaining 34 interviews was 29 min: range 15 to 60 min. There were 20 female and 14 male participants, with a mean age of 67 years: range 51 to 96 years (Table 2).

**TABLE 2:** Participant demographics, most bothersome symptom and consultation status

ID^a^	Age	Sex	Marital status^b^	Socio‐economic decile^c^	Most bothersome symptom	Consulted family doctor?^d^
13863	76	F	Married/cohabiting (Mar/co)	8	Persistent diarrhoea	Yes
14137	56	F	Mar/co	8	Indigestion	No
14330	78	F	No longer	8	Tired all the time	Yes
14921	62	F	Mar/co	6	Altered bowel habit	No
14978	70	F	No longer	6	Altered bowel habit	Yes
16647	64	M	Mar/co	8	Unexplained weight loss	Yes
18633	66	M	No longer	10	Persistent cough	Yes
18709	65	F	Mar/co	5	Persistent heartburn	Yes
20012	79	M	Mar/co	7	Coughing up phlegm	No
20127	61	F	Mar/co	7	Difficulty swallowing	Yes
20979	55	F	Mar/co	8	Abdominal pain	Yes
21020	51	F	Mar/co	7	Abdominal pain	Yes
21033	51	F	Mar/co	5	Breast lump	Yes
26207	73	M	Mar/co	8	Persistent vomiting	Yes
28549	67	F	Single	1	Coughing up phlegm	Yes
28690	61	M	No longer	1	Heartburn	No
31416	68	M	Mar/co	5	Persistent cough	No
32459	60	M	Mar/co	8	Rectal bleeding	Yes
33700	78	F	Mar/co	9	Persistent constipation	No
34798	74	F	Mar/co	3	Persistent vomiting	Yes
41783	84	F	No longer	10	Change in bowel habit	Yes
42737	55	M	Mar/co	8	Difficulty swallowing	Yes
43838	67	F	Mar/co	4	Chest pain	Yes
44016	73	F	Single	5	Shortness of breath	No
46300	67	M	Mar/co	5	Chest pain & breast lump	Yes
49014	55	F	Mar/co	3	Abdominal pain	No
49463	70	M	Mar/co	3	Abdominal pain	Yes
49779	57	M	No longer	2	Shortness of breath	Yes
52190	69	M	Single	9	Loss of appetite	No
53580	61	F	Mar/co	5	Tired all the time	Yes
55000	64	F	Mar/co	7	Rectal bleeding	Yes
58757	79	M	Mar/co	8	Persistent cough	Yes
59379	96	M	Mar/co	7	Tired all the time	Yes
59394	58	F	Mar/co	7	Hoarseness	No

^a^ Participant ID is a unique five‐digit code and gives no indication as to the order of the interviews.

^b^ Split into three categories: married/civil partnership or cohabiting (Mar/co) and no longer living with partner (No longer), which includes divorced, separated and widowed; and the third category is single.

^c^ Socio‐economic status is indicated as a decile where 1 is most deprived and 10 is most affluent. For Scottish patients, the Scottish Government's Index of Multiple Deprivation 2012 was used, and for English patients, the UK Government's Indices of deprivation 2015 was used. Both are similar and have not been differentiated here.

^d^ Whether the participant had consulted the family doctor (GP) about their symptom, as reported in the original USEFUL questionnaire survey.

Themes are presented below and additional supporting quotations are presented in Table S1.

### 3.1 Theme 1: Network activation to establish frames of reference

Participants recognised that it is normal to experience some symptoms and that normality can change with advancing age and stage of life, for example, during the menopause. Participants looked to family members to establish whether there might be a genetic predisposition to cancer and a range of non‐cancer conditions (e.g., stomach ulcers and thyroid disease) that might explain their symptom(s). They compared themselves to other people to establish what was normal at a given age. Sometimes this comparison process was conducted explicitly through conversations with family or friends (Table S1).

In other cases, comparisons were made using the participant's knowledge of others' activities and habits. For example, one 78‐year‐old lady who felt tired all the time noted that her 83‐year‐old sister was still playing tennis. Comparisons were also made within the wider social environment:

I notice also that it could be an age thing as well. I have noticed a lot of older people do clear their throat. I've become aware of that, because I've got it myself now, that I have become aware (…) maybe my generation, it seems to be kind of common. (Participant 28549, 67‐year‐old female, coughing up phlegm)

Downward comparison, that is, the perception that others are worse off, was reported. For example, one participant with a persistent cough compared herself to her sister who was perceived as having more ‘serious’ health problems: ‘compared to her I have nothing’. Downward comparison could lead to a reluctance to mention symptoms to peers (particularly those with health problems). It could also lead to a reluctance to ‘bother’ the doctor because the participant's complaints were deemed more trivial than those of others.

### 3.2 Theme 2: Variation in network activation

Network activation varied based on who was available and the nature or closeness of the relationship. Those who lived alone or had limited social networks were at risk of becoming more isolated if symptoms interfered with usual activities.

Symptom type was an important modifier of network activation. There were subjective norms about the types of problems that could be discussed within different networks and a range of symptoms (e.g., shortness of breath, rectal bleeding and diarrhoea) that participants found too embarrassing to discuss with more distant friends and acquaintances:

It's just my husband and myself. So I mean I talk to him about [diarrhoea]. There's nobody else to talk to about it. And it's not the kind of subject that you raise at the painting group. There are friends individually that I could have talked to about it, but you know, it's not the most fragrant of subjects to go into (…) I used to teach sex education (…) masturbation is one thing that nobody ever wants to talk about. And I think diarrhoea is another one. (Participant 13863, 76‐year‐old female, diarrhoea)

Symptoms were more likely to be raised with friends and family if they were noticeable or impacted upon social activities and were usually raised in order to justify or explain altered behaviour or activities. Examples included diarrhoea that necessitated excusal to the toilet, and dysphagia, cough or appetite loss that affected shared meals (Table S1).

The dynamic circumstances of interpersonal relationships influenced personal network activation, with recognition that symptom disclosure could affect others. Participants were mindful of competing priorities in others' lives, such as the presence of illness, or happy occasions, such as the birth of a child. There was a desire to protect loved ones from worry.

### 3.3 Theme 3: The socio‐cultural context of network activation for potential cancer symptoms

Although cancer was not referred to in any of the study materials or by the interviewer, participants frequently mentioned cancer. Ten of the participants mentioned cancer concerns when completing the original questionnaire and 22 explicitly raised cancer during their interview, with another implying that cancer (and asbestos exposure) had been a concern. Cancer was a disease that was feared.

Participants frequently mentioned knowing somebody within their family or social network with cancer. The experiences and narratives of others directly influenced how participants responded to their symptoms:

I lost a friend with …. she had cervical cancer and then it spread. And what she was telling me, she shared her symptoms with me (that was over 5 years ago), and then the symptoms that my dad had, because he died of bowel cancer, and I just thought, ‘Oh.’. I just wondered if there was something there, you know. So I just thought I'd go and get it checked out. (Participant 49014, 54‐year‐old female, abdominal pain)

Many participants searched the internet to research their symptoms, and cancer was prominent online. Participants also referred to media campaigns about cancer and the fact that cancer was in the public psyche. Specific advertising campaigns, leaflets within healthcare premises, television and radio programmes were all mentioned (Table S1).

There was a strong sense of the association between unhealthy behaviour and disease. Mental representations of cancer (referred to as ‘prototypes’ in the Common‐Sense Model) tended to involve smoking, alcohol, unhealthy diet and feeling seriously unwell. Not identifying with, or fitting, the cancer prototype could inhibit help‐seeking (Table S1).

### 3.4 Theme four: Consequences of network activation

Personal network activation tended to prompt an active coping response to a symptom. Other people offered ‘nuggets’ of information or facilitated information gathering. Others were consulted to gauge the appropriate initial response to a symptom:

we [female friend] talked about the rationale of, you know, what I was thinking, why I was thinking it, and whether it made sense to, you know, do nothing initially and then, you know, at what point there might be a chance to ask questions (…) I think she seemed to think she would do the same thing. It was a sounding board. (Participant 21033, 51‐year‐old female, breast lump)

Other people suggested potential diagnoses for symptoms based upon their own experiences or recommended medicines to try (Table S1).

There were moral dimensions to symptom disclosure. Participants were critical of others with similar symptoms which were perceived to be due to lifestyle choices (for example, heartburn associated with dietary factors or obesity): One participant was told by his daughter that his rectal bleeding could be caused by poor diet (Table S1).

Network activation could lead to judgement by others. One participant speculated that discussing his heartburn with his sister would lead to advice to cut down on smoking. An individual with a persistent cough noted that many of his peers had also experienced coughs and had been dismissive of discussions about symptoms (Table S1).

There were no examples of participants being directly discouraged from seeking help by others. Social network activation was often pivotal in making the decision to seek help. One participant decided to seek help when work colleagues remarked about the persistent nature of her cough. In several cases, a spouse or close family member directly advised the participant to see their GP. Family members could be even more directive, taking action for the participant:

My daughter‐in‐law saw me taking the sheets to wash, and she had me down at, you know, Emergency—which I thought was overdoing it a bit, but you don't argue with my daughter‐in‐law. (Participant 13863, 76‐year‐old female, diarrhoea)

## 4 DISCUSSION

### 4.1 Main findings

Social network activation is an important component of symptom appraisal and the actions subsequently taken. Network activation takes many forms, from direct conversations with others to more subtle comparisons with peers and wider society. When others become directly involved, they seem more likely to prompt an active coping response, for example, encouraging medication use or, more often, suggesting that the individual seek professional help. This study has identified practical, attitudinal, emotional, normative and moral considerations that affect whether and how personal network activation occurs. Factors such as social isolation, embarrassment and stigma about the role of lifestyle in causing symptoms can prevent personal network activation and could impede professional help‐seeking.

### 4.2 Comparison with existing literature

This study adds granularity to existing knowledge about the importance of social networks in cancer symptom appraisal. Specific factors have been identified that influence network activation. Despite a wealth of sociological research examining help‐seeking, few studies have focused specifically on symptoms potentially indicative of cancer. In the cancer literature, studies about symptom appraisal have mainly involved patients with a cancer diagnosis, potentially introducing recall bias. This ethically approved study ‘masked’ our interest in cancer, while exploring help‐seeking for potential cancer symptoms in depth.

Participants in this study looked to others as frames of reference to determine what is normal. They made age‐appropriate comparisons with peers or wider society. Fayers and Sprangers (2002) demonstrated that individuals use various peer group comparisons as frames of reference when they respond to questions about overall health and quality of life. Fayers et al. (2007) observed that some patients enlist a reference group of healthy peers, whereas others use people who are ill when making their comparisons. A ‘downward comparison’ process has been noted, in which individuals enhance their own health status by comparing themselves to others who are worse off (Fayers et al., 2007; Wills, 1981). It is feasible that downward comparison could normalise or trivialise symptoms of potential cancer. Downward comparison might also feed into moral judgements about seeking help in a resource limited healthcare system.

Identifying with someone else's cancer narrative was a strong driver of action after experiencing a potential cancer symptom. Macdonald et al. (2019) explored publicly recognised shared cancer narratives in individuals with colorectal cancer and identified that cancer narratives are dominated by ‘fear, death, and severity’. We found that media campaigns have contributed to public awareness of a variety of cancer symptoms, including less ‘alarming’ ones such as persistent cough. We also noted a tension between campaigns that target risk factor modification in healthy individuals and those that target help‐seeking in symptomatic individuals. When these public narratives are combined, a ‘prototype’ or mental representation of cancer is formed which describes a severe illness affecting individuals with unhealthy lifestyles. Failing to identify with such a prototype could delay help‐seeking in some individuals.

The notion of cancer ‘prototypes’ overlaps with concepts of candidacy. In a study exploring public understanding of heart disease, Davison et al. (1991) proposed that candidacy involved an individual's estimation of their disease risk, informed by ‘lay epidemiology’. For example, individuals might consider an overweight smoker to be a ‘candidate’ for heart disease. Lay epidemiology is informed by publicly available information and social experiences (Davison et al., 1991).

Candidacy has been identified as a promising framework on which to base cancer prevention campaigns (Batchelor et al., 2021). Our study suggests that cancer candidacy may also be a useful framework on which to modify individual responses to *symptoms*. Helping individuals to differentiate between risk factors for cancer at the population level, and risk of cancer when symptoms occur is an important avenue that deserves further attention.

### 4.3 Methodological considerations

All participants were sampled from the United Kingdom and reflected upon their experiences within the National Health Service, a ‘gatekeeper’ system. A diverse group of individuals were included, with a wide range of current or recent symptoms and help‐seeking responses to them. Multiple authors with different disciplinary backgrounds were involved in data analysis to reduce the risk of bias. There is debate over the concept of data saturation (Braun & Clarke, 2021; Low, 2019). We relied upon the interviewer to judge when saturation had occurred and found that 34 interviews were sufficient to fully explore the topic.

The study was undertaken prior to the COVID‐19 pandemic. Arguably, findings that social isolation and barriers to personal network activation modify individual responses to potential cancer symptoms are particularly salient in the current context of unprecedented, enforced social restrictions.

Several limitations apply. The sampling frame was derived from respondents to a questionnaire that only included individuals aged 50 and over. Results may not reflect those of a younger age group; however, the symptoms that were investigated are more likely to be cancer‐relevant in an older age group. Many participants used the internet to find information when appraising their symptoms. None mentioned engaging with social networks online or via sites such as Facebook or Twitter. Further research is required to understand the potential role of online social networks, and it may be that younger individuals make greater use of online social networking sites. Participants were able to discuss multiple symptoms during the interviews, but there was a particular focus on the most bothersome symptom. There is scope to examine how the interplay of multiple symptoms might affect help‐seeking in subsequent analyses. We have not examined whether there are differences in experiences or views based on certain demographics such as age or socio‐economic status, and we were not able to purposively sample based on ethnicity because ethnicity data were poorly completed in the original questionnaire.

## 5 CONCLUSIONS

Social networks and wider society have an important influence on how individuals respond to symptoms. Decisions about whether to engage social networks are not straightforward; there are practical, psychological and social barriers to involving others. Media campaigns contribute to socio‐cultural knowledge about cancer symptoms and perceived norms about help‐seeking. Tailoring these campaigns to incorporate age‐appropriate narratives, to tackle misconceptions about cancer prototypes and to reduce stigma surrounding lifestyle factors could facilitate early help‐seeking for potential cancer symptoms.

## CONFLICT OF INTEREST

The authors declare that they have no conflicts of interest.

## Supporting information


**Table S1:** Supporting participant quotations

## Data Availability

The data that support the findings of this study are available on request from the corresponding author. The data are not publicly available due to privacy or ethical restrictions.
