# Supplementary material for: How does social context influence appraisal and help‐seeking for potential cancer symptoms in adults aged 50 and over? A qualitative interview study
Source: Eur J Cancer Care (Engl). 2022 Jun 21;31(6):e13640. doi: 10.1111/ecc.13640 (PMC9787863; doi:10.1111/ecc.13640)
Supplement: Supplementary file 1 — Table S1: Supporting participant quotations [file ECC-31-e13640-s001.docx]

**Table 3: Supporting participant quotations**

| Theme one: network activation to establish frames of reference | Explicit comparisons with family and friends | “It’s talking to people really. Like friends when I go out for lunch with, and we’re all sort of saying, ‘Oh my God, we’re 60 and this is happening, and that’s happening. And other people seem to be getting this tiredness. So, maybe we’re expecting our bodies to do more than they should at this age.” Participant 53580. 60-year old female, tired all the time. |
| --- | --- | --- |
| Theme two: variation in network activation | Symptoms raised with friends and family in order to explain altered behaviour or activities | “I think the only circumstances where I’ve discussed it [swallowing difficulty] with people outside of the family circuit would be, I can think of a couple of occasions socially and in business where maybe I’ve been at a business lunch and the symptoms have occurred and I have to excuse myself. And then I’ll simply say to the… briefly explain the condition to the people involved. So it’s not something I’d ever offer as a subject of conversation, I more offer it up by way of explanation, when I’ve excused myself from the dinner table”. Participant 42737, 55 year old male, difficulty swallowing. |
| Theme three: the socio-cultural context of network activation for potential cancer symptoms | Media campaigns about cancer: cancer is in the public psyche | “I think because of the publicity that you hear, or read, you know, it made me stop and think, and made me make the decision that these things can’t be left, that you’ve got to go and get them investigated, regardless [of] what that might involve and whether you find it unpleasant or embarrassing, or whatever, it was something that really needed to, to be looked at, and sooner rather than later.” Participant 55000, 64-year old female, rectal bleeding: |
|  | Cancer prototypes – cancer as a disease that affects individuals with unhealthy lifestyles. Cancer involves feeling seriously unwell. | “I wasn’t really too concerned, because I don’t smoke, I’m quite healthy, I don’t really drink (…) I’ve always been healthy, I’ve got great health. So I didn’t have any reason to, you know (… ) I think it was three months I’d had it, and the minute they see a cough as long as that, I know the doctors become concerned - because I’ve read that in the papers and I’ve seen it on the television (…) I think, because of the awareness now of cancer, you know. But I wasn’t kind of concerned, to be honest with you because I felt okay.” Participant 28549, 67-year old female, coughing up phlegm. |
| Theme four: consequences of network activation | Other people offered medicines to try | “A friend who comes Saturday night, and we have a drink together. You know, I kept saying, ‘Oh I’ve got this… I’ve got this horrible pain.. He says, ‘Hang on, I’ve left some stuff in the van, Buscopan stuff. And he brought it in and I had a swig of that, and it went a bit easier. Because he suffers from heartburn and indigestion himself”. Participant 49463, 70-year old male, abdominal pain |
|  | Moral dimensions to symptom disclosure: judgement about diet | “my daughter was saying, well it’s maybe what you’re eating, you know. You’re eating… she comes out with the words rubbish, shit, or whatever… garbage.” Participant 32459, 60 year old male, rectal bleeding |
|  | Friends can be dismissive of symptom discussions | “I’ve spoken to friends, and say, ‘Oh my, I’ve got a bad cough here.’ (…) Let’s say I was out having a meal with them or something of that order and I’d say, ‘I’ve got this rotten cough’ or ‘this awful cough’ (…) And they would just look at me and say, ‘Well yes, I’ve got one as well.’ Participant 58757, 79 year old male, persistent cough |
